# Detailed reconstruction of the musculature in *Limnognathia maerski* (Micrognathozoa) and comparison with other Gnathifera

**DOI:** 10.1186/s12983-014-0071-z

**Published:** 2014-10-01

**Authors:** Nicolas Bekkouche, Reinhardt M Kristensen, Andreas Hejnol, Martin V Sørensen, Katrine Worsaae

**Affiliations:** Marine Biological Section, Department of Biology, University of Copenhagen, Universitetsparken 4, 2100 Copenhagen Ø, Denmark; Natural History Museum of Denmark, Universitetsparken 15, 2100 Copenhagen Ø, Denmark; Sars International Centre for Marine Molecular Biology, University of Bergen, Thormøhlensgate 55, Bergen, N-5008 Norway; Natural History Museum of Denmark, Øster Voldgade 5-7, 1350 Copenhagen K, Denmark

**Keywords:** CLSM, 3D reconstructions, Jaw apparatus, F-actin, Trophi, Mastax

## Abstract

**Introduction:**

*Limnognathia maerski* is the single species of the recently described taxon, Micrognathozoa. The most conspicuous character of this animal is the complex set of jaws, which resembles an even more intricate version of the trophi of Rotifera and the jaws of Gnathostomulida. Whereas the jaws of *Limnognathia maerski* previously have been subject to close examinations, the related musculature and other organ systems are far less studied. Here we provide a detailed study of the body and jaw musculature of *Limnognathia maerski*, employing confocal laser scanning microscopy of phalloidin stained musculature as well as transmission electron microscopy (TEM).

**Results:**

This study reveals a complex body wall musculature, comprising six pairs of main longitudinal muscles and 13 pairs of trunk dorso-ventral muscles. Most longitudinal muscles span the length of the body and some fibers even branch off and continue anteriorly into the head and posteriorly into the abdomen, forming a complex musculature. The musculature of the jaw apparatus shows several pairs of striated muscles largely related to the fibularium and the main jaws. The jaw articulation and function of major and minor muscle pairs are discussed. No circular muscles or intestinal musculature have been found, but some newly discovered muscles may supply the anal opening.

**Conclusions:**

The organization in *Limnognathia maerski* of the longitudinal and dorso-ventral muscle bundles in a loose grid is more similar to the organization found in rotifers rather than gnathostomulids. Although the dorso-ventral musculature is probably not homologous to the circular muscles of rotifers, a similar function in body extension is suggested. Additionally, a functional comparison between the jaw musculature of *Limnognathia maerski*, Rotifera and Gnathostomulida, emphasizes the important role of the fibularium in *Limnognathia maerski*, and suggests a closer functional resemblance to the jaw organization in Rotifera.

## Introduction

*Limnognathia maerski* Kristensen & Funch, 2000, is a minute animal living in fresh water ponds and lakes [1–3]. The animal was discovered in 1994 at Disko Island, Greenland, but not described before 2000, and it has subsequently been reported from the Sub Antarctic Crozet Island [1], in a stream from southern Wales, United Kingdom, and in the river Lambourn (Berkshire), United Kingdom (P. E. Schmid and J.M. Schmid-Araya, personal communication). With a unique combination of characters, it is considered the only member of the recently described Micrognathozoa [2–5], belonging to Gnathifera. However, the phylogenetic relationships within Gnathifera are still debated, and the molecular studies are based on very limited information [5]. So far, the complex jaw apparatus of *L. maerski* has received the main attention in studies, leading to several disputed homology hypotheses for each sclerite of the trophi [1,3,6,7]. However, no detailed studies have addressed the overall morphology of organs systems and further anatomical knowledge on *L. maerski* is warranted in order to compare this unique evolutionary lineage with the other gnathiferan groups, as well as other animals.

*Limnognathia maerski* measures 80-150 μm, possesses a complex set of jaws, a conspicuously arranged ventral ciliation and, so far, only females are known. The ventrally ciliated head consists of a forehead with ciliary sensory organs and a more posterior part containing the pharyngeal apparatus. The trunk is composed of an accordion-like thorax and a large abdomen with ventral ciliophores and a posterior adhesive pad [3]. In the original description, the overall musculature of *L. maerski* is briefly described. It is composed of several longitudinal and dorso-ventral muscles, minute muscles articulating the dorsal plates and a dense pharyngeal musculature. No circular musculature has been found. However, precise information on the number, configuration and relative size of each set of muscles was not provided. Ultrastructural data provided information on the structure of muscles attachment sites, the absence of myosyncytia and myoepithelia, the cross-striated nature of the pharyngeal musculature, and the mainly obliquely striated longitudinal musculature [3].

Following Sørensen [6], the jaws of *L. maerski* are composed of six main elements: i) The median, ventral-most basal plate with posterior stems and anterior flattened and toothed manus, ii) the large and conspicuous ventral fibularium made of different chambers containing cells, extending dorso-laterally, iii) the latero-ventral ventral jaws (pseudophalangia) that articulate posteriorly with the associated accessory sclerites, iv) the medio-dorsal main jaws, each with a posteriorly projecting cauda, surrounded by the fibularium, v) the dorso-lateral dorsal jaws also confined to the fibularium area, vi) and the pharyngeal lamellae, a pair of lamellate structures positioned antero-laterally to the rest of the jaw apparatus. Additionally, Kristensen and Funch [3], describe the lamella orales as a paired structure similar to the lamellae pharyngea, situated dorso-laterally, inside the fibularium. However, the presence of these structures has not been confirmed in any subsequent studies [1,6].

**Figure 1:**
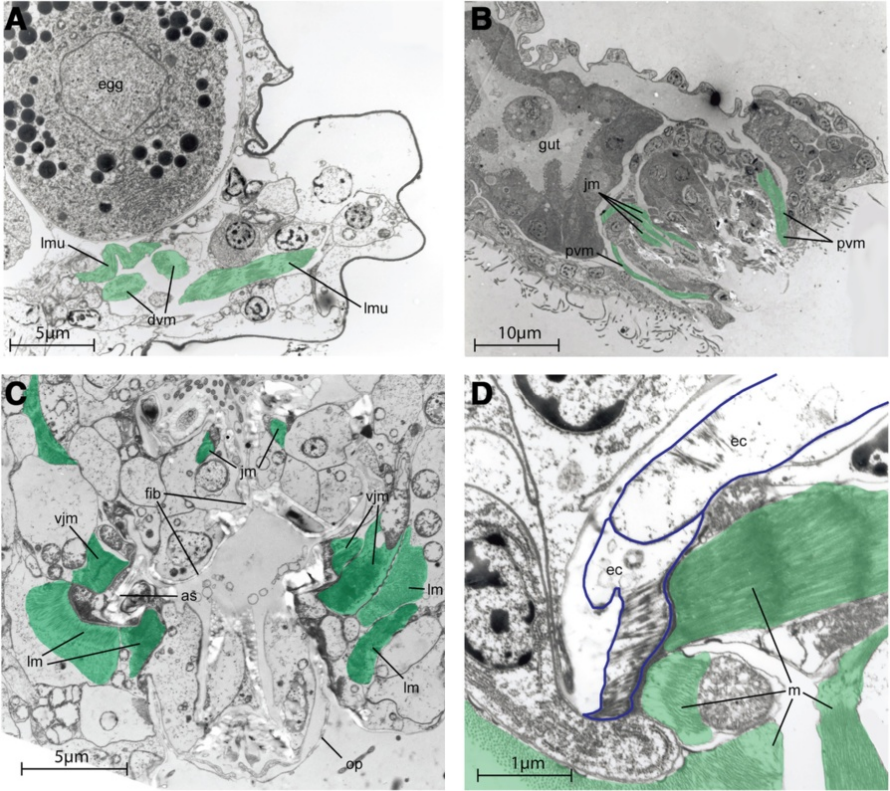
**TEM sections of** ***Limnognathia maerski*** **.** Muscles highlighted in green. **A**, transversal section of posterior part. Posterior on the right. **B**, sagittal section showing the vomit behaviour. **C**, transversal section of the jaws. The ventral side is on the bottom. **D**, Close up of muscle attachment on a jaw sclerite, showing the non myoepithelial nature of the jaw muscles. Epidermal cells with blue outlines.

The animal lives in limnic mosses or in the sediment of relatively calm springs and lakes, and was first recognized for its unusual ‘ciliate-like’ swimming in the water column. It also uses ciliary motion to glide over surfaces. Occasionally, it performs muscular contractions during lateral bending and longitudinal accordion like contractions for directional change, ventral bending while egg laying and dorsal contraction during vomit behavior [3]. Foraging of *L. maerski* involves fine movements of the jaw apparatus as well as larger movements of the head. While feeding, the ventral jaws are protruded and involved in substrate grasping. During the vomit behavior, the forehead is moved upward and backward, and most of the jaw apparatus is protruded through the mouth opening, while it performs fast snapping movements of the jaw elements and forward and backward movements of the main jaws (see reference [3] and Figure 1B). Accessory sclerites and pseudophalangia may move independent of the rest of the jaw apparatus, allowing the ventral jaws to move from a rostro-caudal orientation to a dorso-ventral orientation without moving the other jaws elements [3,6,7].

The body wall musculature differs between the putatively closest micrognathozoan relatives: Gnathostomulida and Rotifera. In Gnathostomulida, the overall musculature consists of numerous circular and diagonal muscles and several bundles of longitudinal muscles (six to nine pairs [8–10]) extending the entire body length, where the superimposition of longitudinal, diagonal and circular muscles forms a dense grid like body wall musculature [9,10]. In the majority of rotifers, most of the longitudinal muscles do not extend through the entire body, but are limited to certain body regions, e.g., coronal retractors in the head or muscles in the posterior part of the trunk, being involved in the contraction of the head and foot, respectively [11–13]. Circular muscles are few and usually incomplete transverse, rather than circular (*e.g.,* [11–15]), although some Gnesiotrocha have complete rings [12,16,17]. Most of the diagonal and transverse muscles are usually absent (*e.g.*, [12,18]), and if present they are only few and/or inconspicuous [14,19]. Splanchnic muscles surrounding the gut are not found in Gnathostomulida [9], whereas they present a very thin musculature in Rotifera. This muscular grid is documented for Seisonidae [11] and Monogononta (*e.g.*, [13,19]) but visceral muscles are not found in Bdelloidea [12,14]. Dorso-ventral musculature has not been described for Gnathostomulida [9], and most of the functionally dorso-ventral muscles in Rotifera are supposedly modified incomplete circular muscles [12,19] meaning “true” dorso-ventral muscles, as reported by Kristensen and Funch [3], seem to be unique for Micrognathozoa.

The jaw musculature also differs between Gnathostomulida and Rotifera, due to the organization of their jaws. In gnathostomulids, the jaw apparatus consists of i) a set of main jaws, and in some taxa ii) an unpaired basal plate [20–22]. In rotifers, the jaw apparatus (trophi) includes 7 main elements: the i) unpaired posteriorly directed fulcrum, ii) paired rami, iii) paired unci, and iv) paired manubria. The fulcrum and rami together form the central element, the incus, whilst the unci and manubria form the mallei (*e.g.*, [23–25]). The rotifer incus has been considered homologous with the gnathostomulid main jaws [21,26]. However, it also has been suggested that some parts of the gnathostomulid articularium (antero-lateral parts of the main jaws) are homologous with the rotifer manubria [27]. The gnathostomulid basal plate is considered autapomorphic for the group, and no homologous counterpart has been identified in the rotiferan trophi. The structural differences in the musculature of gnathostomulid and rotiferan jaw apparatuses clearly relate to the differences in the hard parts and the additional number of rotifer jaw elements. Indeed, most of the musculature supplying the rotifer trophi consists of relatively small paired muscles connecting the different jaw elements (sclerites), while, in Gnathostomulida, the main jaws are mainly moved together by large muscles attached to the pharynx wall. The movement between jaw elements in Gnathostomulida is consequently achieved by U-shaped muscles (bent transversal muscles) and laterally attached transversal muscles.

Recently, several CLSM studies of phalloidin-stained musculature have been carried out on a great number of microscopic animals, revealing comprehensive information on their overall musculature [9,11,28–30] and also, in the case of gnathiferans, on the musculature of the rotifer mastax [31,32] or gnathostomulid pharynx [26]. Combined with TEM, many details can be inferred on the relative position of muscles and their ultrastructure, but also connections to the other part of the body. In order to compare the general muscular organization as well as jaw musculature of *L. maerski* with other animals, we here describe its musculature employing F-actin staining and confocal laser microscopy (CLSM) as well as transmission electron microscopy (TEM).

## Results

**Figure 2:**
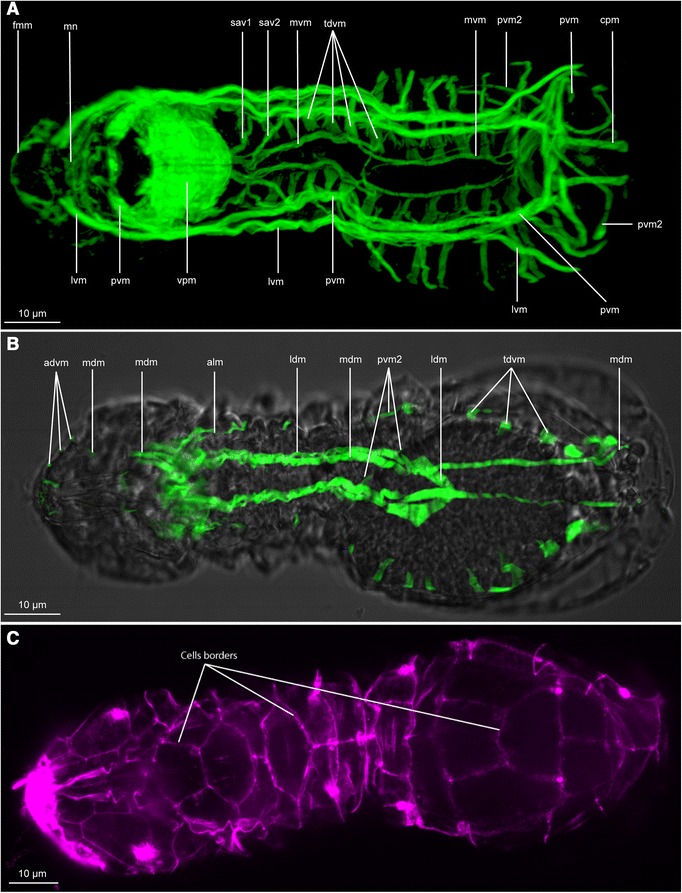
**CLSM of phalloidin stained muscle system and light microscopy of** ***Limnognathia maerski*** **.** Anterior end is positioned left on all pictures. **A**: Ventral view, Z-stack of the ventral portion, showing only the muscle system. **B**: Single section showing CLSM of the dorsal muscle system and the contour of the specimen, visualized with transmitted light. **C**: Synapsin2 staining of *L. maerski*, maximum intensity projection of a dorsal substack. Lines show the border of the dorsal cells to which the dorso-ventral muscles attach (illustrated in Figure 4B). advm, anterior dorso-ventral muscles; alm, anterior lateral muscle; cpm, ciliated adhesive pad muscle; fmm, front margin muscle; ldm, lateral dorsal muscle; lvm, lateral ventral muscle; mdm, median-dorsal muscle; mvm, medio-ventral muscle; mn, muscle network; pvm, paramedian ventral muscle; pvm2, posterior lateral muscle; sav1,2, small anterior ventral longitudinal muscles; tdvm, trunk dorso-ventral muscles; vpm, ventral pharyngeal muscles.

**Figure 3:**
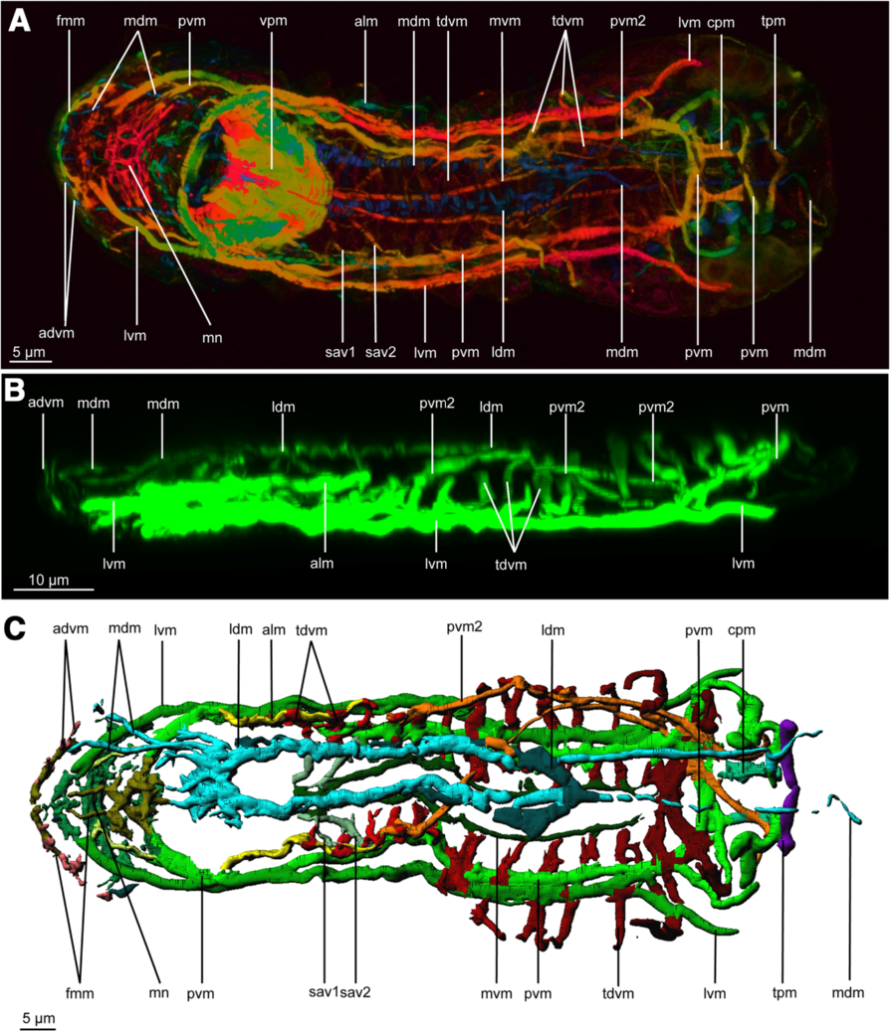
**CLSM of phalloidin stained muscle system of** ***Limnognathia maerski*** **.** Anterior end is positioned left on all pictures. **A**, Ventral view of the maximum depth intensity projection. **B**, lateral view reconstruction of a dorso-ventral Z-stack. Same specimen as Figure 2A,B. **C**, Dorsal view of the isosurface reconstruction of the muscular system. Same specimen as Figure 2A,B. advm, anterior dorso-ventral muscles; alm, anterior lateral muscle; cpm, ciliated pad muscle; fmm, front margin muscles; ldm, lateral dorsal muscle; lvm, Lateral ventral muscle; mdm, medio-dorsal muscle; mn, muscle network; mvm, medio-ventral muscle; pvm, paramedian ventral muscle; pvm2, posterior lateral muscle; sav1,2, small anterior ventral longitudinal muscles; tdvm, trunk dorso-ventral muscles; tpm, transversal posterior muscle; vpm, ventral pharyngeal muscles.

**Figure 4:**
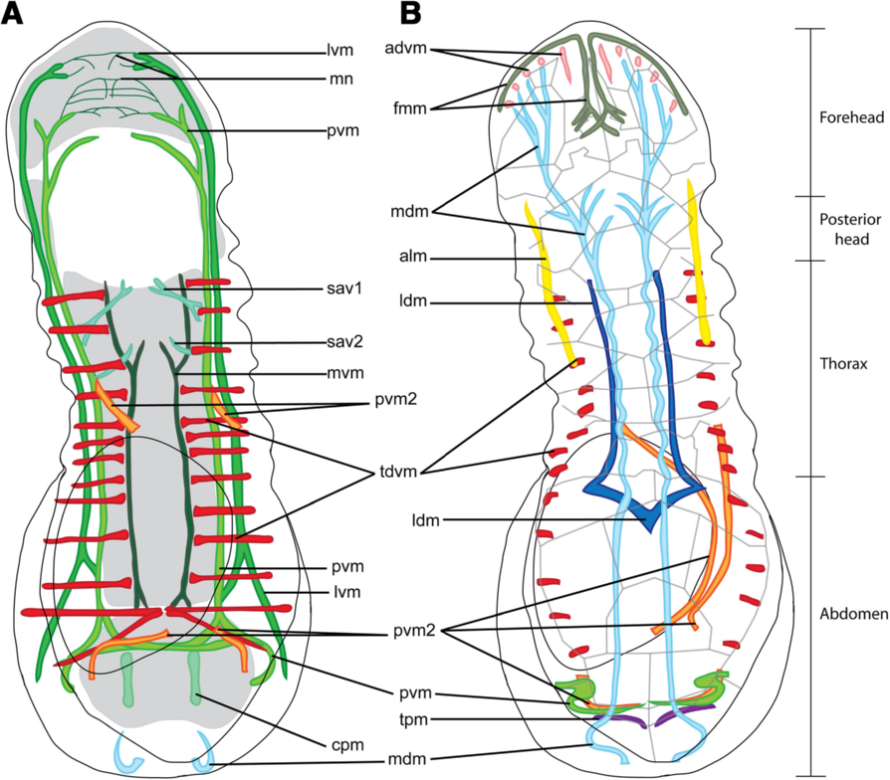
**Schematic drawings of the somatic musculature of** ***Limnognathia. maerski*** **.** Anterior is on the top. Colors follow Figure 3C. **A)** Dorsal view of the ventral musculature (colors) relative to body wall and ciliated areas (grey shade). **B)** Dorsal view of the dorsal musculature (colors) and its attachment sites on dorsal epidermis cells (delimitated in light grey) attachment sites of anterior 5 trunk dorso-ventral muscles are inferred. The mdm, pvm, pvm2, and tdvm are present in **A)** and **B)** as they extend ventrally and dorsally. advm, anterior dorso-ventral muscles; alm, anterior lateral muscle; cpm, ciliated adhesive pad muscles; fmm, front margin muscle; ldm, lateral dorsal muscle; lvm, lateral ventral muscle; mdm, medio-dorsal muscle; mn, muscle net; mvm, medio-ventral muscle; pvm, paramedian ventral muscle; pvm2, paramedian ventral muscle 2; sav1,2, small anterior longitudinal muscle; tdvm, trunk dorso-ventral muscle; tdm; trunk posterior-muscle.

**Figure 5:**
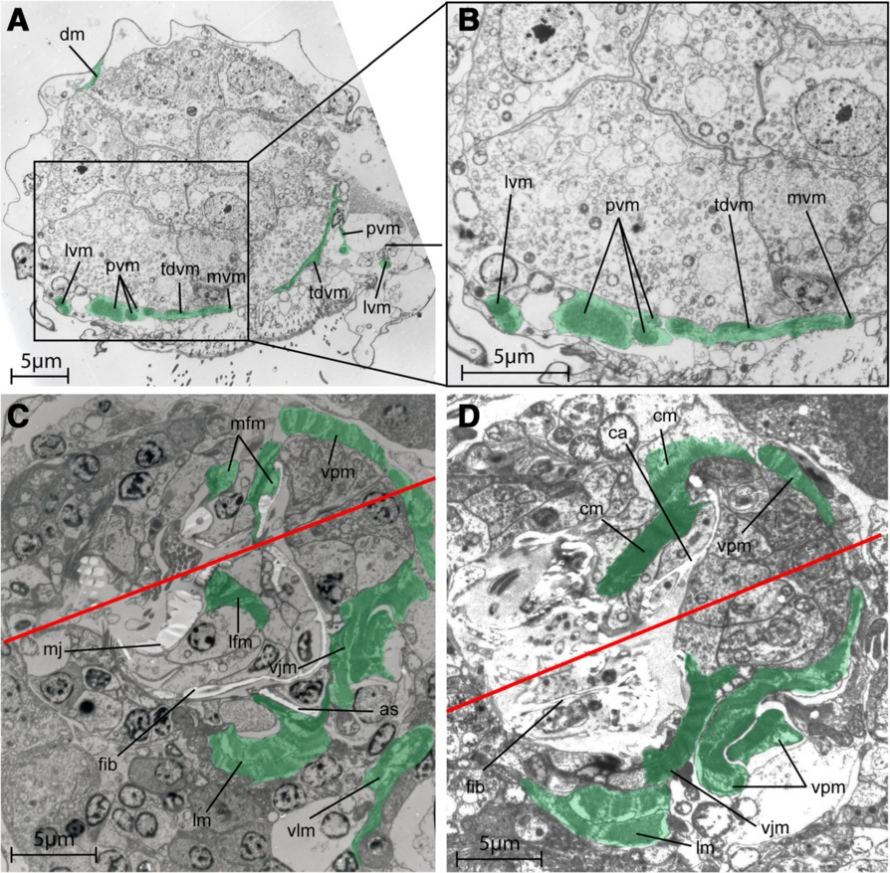
**TEM sections of** ***Limnognathia maerski*** **.** Muscles highlighted in green. **A**, transversal section of the trunk*.* Dorsal side on top. **B**, close up of figure A., showing the ventral musculature. **C, D**, coronal section the jaws. The red line shows the symmetry axis of the jaws. The front is on the left. The section in C is more ventral than the section in D. as, accessory sclerite; dm, dorsal muscle; ca, cauda; cm, cauda muscle; fib, fibularium; lfm, lateral fibularium main jaw muscle; lm, pharyngeal lamella muscle; lvm, lateral ventral muscle; mfm, median fibularium main jaw muscle; mj, main jaws; mvm, medio-ventral muscle; pvm, paramedian ventral muscle; tdvm, trunk dorso-ventral muscle; vjm, ventral jaw muscle; vlm, ventral lateral muscle; vpm, ventral pharyngeal muscle.

The overall musculature is organised into seven main pairs of longitudinal muscles extending from head to abdomen and 13 oblique dorso-ventral muscles localised in the thoracic and the abdominal part (Figures 2, 3, and 4). No circular muscles are present. The musculature furthermore comprises the dense pharyngeal muscle and the fine anterior forehead muscle. Cross striated muscles are found in the body wall musculature (Figure 1A) as well as in the jaw musculature (Figures 1B,C,D and 5C,D).

### Longitudinal musculature

The longitudinal musculature of the trunk consists of seven pairs of main muscles (*three ventral, two lateral, two dorsal*) as well as two short anterior pairs of muscles and two short posterior pairs of muscles.

#### Ventral muscles

The three ventral main muscles extend the body length aiding the body contraction and extension (Figures 2A, 3 and 4). The longitudinal ventral muscles are implicated in longitudinal contractions and ventral bending.

The paired medio-ventral muscles (mvm, Figures 2A, 3A,C, 4 and 5A,B) consist of two muscle fibres that form bundles originating directly posterior to the ventral pharyngeal muscle and extend along the ventral wall of the gut (mvm: Figure 5A). At its posterior extremity, each medio-ventral muscle separates into two very short muscle fibers that each extends four micrometers before inserting into the epidermis that is anterior of the adhesive ciliated pad (mvm: Figure 4).

Medially, two pairs of small anterior ventral longitudinal muscles (sav1, sav2, Figures 2A, 3A,C and 4) supply the anterior part of the thorax, each originating from the midline directly posterior to the ventral pharyngeal muscle. The anteriormost muscle pair (sav1) is bifurcated at both ends: the anterior bifurcation inserts medially just behind the pharynx, while the posterior bifurcation originates in a more lateral region close to the paramedian ventral muscle (sav1: Figure 4). The posteriormost muscle pair (sav2) inserts medially at the level of mvm and extends laterally toward (and originates close to) the paramedian ventral muscle (described below, sav2: Figure 4).

Latero-anterior to the pharynx are three muscles that come together to form the paramedian ventral muscle (pvm, Figures 2A, 3A,B,C, 4 and 5A,B); consequently, the paramedian ventral muscle is trifurcated at its anterior insertion but extends posteriorly as a single muscle bundle. The paramedian ventral muscle follows the course of the trunk and abdomen, where it eventually bifurcates into two separate bundles. The ipsilateral muscle bundle extends dorsally where it joins the paramedian ventral muscle 2 on the same side of the abdomen, while the contralateral muscle extends to the opposite side of the body and joins the contralateral last dorso-ventral muscle. Thus, each of the last dorso-ventral muscle bundles consists of three separate muscles: a dorso-ventral muscle, an ipsilateral branch of the paramedian ventral muscle and a contralateral branch of the paramedian ventral muscle from the opposite side of the body. (pvm: Figures 2A, 3A, 4 and 5). The paramedian longitudinal muscle follows the outline of the ventral ciliated area and contractions may change the direction during swimming or crawling (pvm: Figure 4).

Each of the two lateral ventral muscles (lvm; Figures 2A, 3A,B,C and 5A,B) inserts anterior of the mouth where they each bifurcate into two smaller branches. Posteriorly, each lateral ventral muscle extends along the trunk and abdomen as a single bundle that eventually bifurcates again. The inner branch joins the paramedian ventral muscle, while the lateral branch inserts in the region of the large posterior gland.

A pair of ciliated adhesive pad muscles (cpm: Figures 2A, 3A,C and 4), which are present as short longitudinal bands, extend from an anterior zone of the ciliated pad (just posterior of the paramedian ventral muscle midline) to a posterior zone of the ciliated pad (cpm: Figure 4). The adhesive ciliated pad muscle is probably involved in the adhesive ciliated pad area contractions. Contraction of the adhesive ciliated pad muscles could contract this area and allow the animal to release from the substratum.

#### Lateral muscles

Two pairs of lateral muscles are present in the trunk. The pair of anterior lateral muscles (alm: Figures 2A, 3B,C and 4B) originates anterior of the mouth, probably bifurcating from the paramedian longitudinal muscle, and continues two thirds into the abdomen, appearing to attach to the lateral epidermal cells. They are positioned at a mid dorso-ventral level. The paired paramedian ventral muscles 2 (pvm2: Figures 2A,B and 3A,B,C) originate ventrally to the paramedian ventral muscles, separating at the mid-thoracic level. Each muscle reaches the dorsal side along the anterior part of the abdomen (pvm2: Figure 2B), extends ventrally at the level of the adhesive ciliated pad and returns at an antero-dorsal position, joining the very posterior dorsal epidermal cells and the paramedian ventral muscle. From this point, both paramedian ventral muscle 2 muscles join close to the midline at their posteriormost point, at the level of the last dorsal plate. If an egg is present at the level of the abdomen, one of the posterior lateral muscles is pushed by the egg to the contralateral side to return to the ipsilateral side at the level of the adhesive ciliated pad (pvm2: Figures 2A, 3A,C and 4). This muscle extends along the dorsal side of the gut, being probably implicated in dorsal bending of the animal.

#### Dorsal muscles

Two dorsal pairs of muscles extend through the trunk. The two pairs are close to the midline and extend as two contiguous muscles (Figures 2B, 3A,C and 4).

The medio-dorsal muscle (mdm: Figures 2B, 3A,B,C and 4. dm: Figure 5) is an elongate band that extends from the head to the abdomen and is composed of several thinner muscles that branch off in the forehead and posterior head regions. Anteriorly, the medio-dorsal muscle branches twice (with additional subbranches) that insert close to the frontal margin where the anterior dorso-ventral muscles insert (advm: Figures 2B, 3A-C and 4). In the posterior head region, the medio-dorsal muscle supplies several short muscle branches just dorsal of the pharynx (mdm: Figures 2B, 3C and 4). At the very posterior part of the animal, the medio-dorsal muscle lines the body wall, to insert at the ventro-posterior extremity of the abdomen (mdm: Figures 2B, 3A,C and 4B).

The lateral dorsal muscles (ldm: Figure 2B, 3A,B, and 4. dm: 5) originate as a pair of muscles that both insert at the midline in the trunk region (mdm: Figure 4). Each muscle extends antero-laterally for about 10 micrometers before curving back medially and continuing anteriorly as a strictly longitudinal muscle band that inserts dorsal to the pharynx (Figures 2B, 3A,C and 4B).

#### Transversal posterior muscles

Additionally, at the very posterior region, a complex of transversal and dorso-ventral muscles is present (Figures 3C and 4). It is partially formed by the longitudinal muscle extending posteriorly, from the ventral to the dorsal side. Posterior of these muscles, two pairs of dorsal small transversal muscles line each side of the body. It is difficult to determine with certitude if these two pairs are the continuity of the posterior lateral muscle. However, the anteriormost pair of lateral muscles seems to be a continuity of the paramedian ventral muscle (pvm: Figures 2A, 3A,C and 4) while the transversal posterior muscle pair seems to be another set of muscles (tpm: Figures 3A,C and 4). Both pairs of transversal posterior muscles are very dorsal and according their anatomical position could be implicated in a possible anus opening. Along with the posterior longitudinal and dorso-ventral musculature, the complex posterior musculature is probably involved in the oviposition, substrate adherence and, eventually, defecation.

### Dorso-ventral musculature

The dorso-ventral musculature consists mostly of two sets of muscles: the anterior dorso-ventral muscles and the trunk dorso-ventral muscles (Figure 3C and 4). The posteriormost dorso-ventral complex is the continuation of the paramedian muscle and the paramedian ventral muscle 2 when they fold in the posterior region, and is not serially homologous to the trunk dorso-ventral muscles.

#### Anterior muscles

Five pairs of anterior dorso-ventral muscles (advm: Figures 2B, 3A,B,C and 4) supply the front margin. They appear to support the frontal ciliated sensory region. On each side, the medianmost dorso-ventral head muscle inserts dorsally, at the anterior head margin, close to the mid-line (Figure 2B).

#### Trunk muscles

Thirteen pairs of oblique trunk dorso-ventral muscles (tdvm: Figures 2A,B, 3A-C; 4; 5A,B) supply the thorax and the abdomen. Each trunk dorso-ventral muscle inserts close to the midline on either side of the medio-ventral muscle, extends laterally dorsal to the paramedian ventral muscle and the lateral ventral muscle, and then curves dorsally to insert on epidermal cells (tdvm: Figures 2A; 4; 5A,B). They join the epidermal cells dorsally, extending along the body sides. They line the gut cells very closely, probably functioning as body-wall musculature as well as gut musculature (tdvm: Figure 5A,B). Five pairs supply the thoracic region and eight supply the abdomen region (tdvm: Figures 2A,B; 3A-C; 4). The penultimate and the last pair of dorso-ventral muscles insert ventrally at the midline where the medio-ventral muscle inserts as well, forming a very muscular zone five micrometres anterior of the adhesive pad. A few micrometres posteriorly, the two paramedian muscles cross transversally, forming with the two last dorso-ventral trunk muscles a triangular set of ventral muscles at the anterior area of the adhesive ciliated pad (tdvm: Figures 2A; 3C; 4).

### Forehead musculature

The head musculature is a continuity of the longitudinal body musculature as well as a few specific muscles.

On the frontal margin, the paired frontal margin muscles (fmm: Figures 2A; 3A,C; 4) follow the coronal plan supplying the anterior ciliated region. The median extremity of each muscle is dorsal and bends posteriorly to continue dorsally as two longitudinal median head muscles. At the distal extremities, the front margin muscles are more ventral and supply the frontal ciliated zone. The five pairs of anterior dorso-ventral muscles also supply the frontal ciliated area. The anterior dorso-ventral muscles extend dorsally and quite close to the frontal margin muscle, thus appearing to be in contact with it. In front of the pharynx, dorsally, a cross like complex of small muscles consists of the front margin muscles continuing as a longitudinal median head muscle and trifurcates as two lateral small bundles and one median bundle. The bundles of the front margin muscles of each side join the midline with other contralateral front margin muscle (fmm: Figures 3C; 4).

Ventro-anteriorly, in front of the mouth opening the continuity of the lateral ventral muscle and the paramedian ventral muscle form a thin muscle network (mn: Figure 2A; 3A,C; 4), probably implicated in some anterior glands or changes of the shape of the head.

### Pharynx musculature

The pharynx musculature includes the major ventral pharyngeal muscle and several paired and unpaired muscles articulating the jaws. Jaw muscles have a non-epidermal origin, with each muscle being connected to an epidermal cell associated to a sclerite (Figure 1D). Thus, the musculature of the jaws is probably of mesodermal origin. The function of the musculature is interpreted according to previous studies on feeding behaviour and live observations.

**Figure 6:**
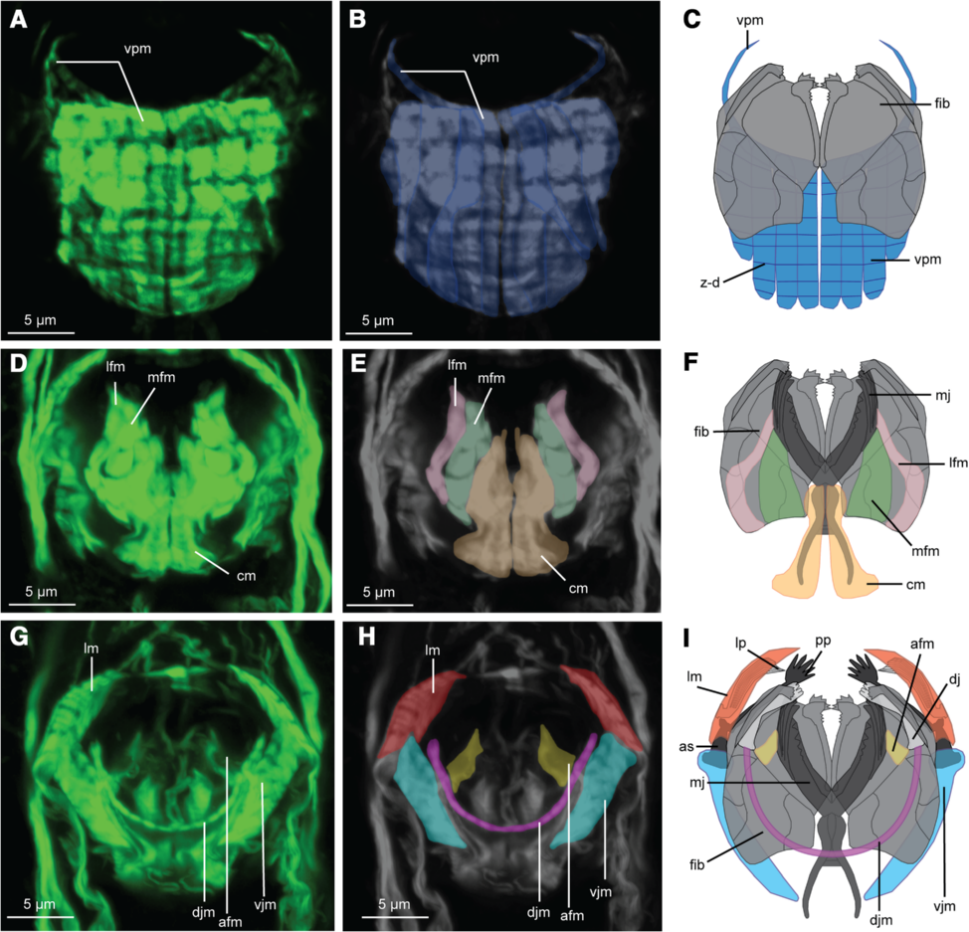
**Musculature and reconstruction of the jaw apparatus of** ***Limnognathia maerski*** **in dorsal view.** Anterior is on the top for all the pictures. **A**, **B**, **C**: ventral part of the jaw system. **D**, **E**, **F**: median part of the jaw system. **G**, **H**, **I**: dorsal part of the jaw system. **A**, **D**, **G**: CLSM of phalloidin stained muscle system, dorsal view of a projection of a sub sample of the Z-stack. **B**, **E**, **H**: enlightenment of the different muscle systems of the jaws. **C**, **F**, **I**: schematic drawing of the dorsal view of the myoanatomy of the jaw system linked to the cuticular elements in greys. Jaw drawing after Sørensen [6]. as: accessory sclerite; afm: anterior fibularium-main jaw muscle; cm: caudal muscle; dj: dorsal jaws; djm: dorsal jaw muscle; fib: fibularium; lm: pharyngeal lamella muscle; lp, pharyngeal lamella; lfm: lateral fibularium-main jaw muscle; mfm: median fibularium-main jaw muscle; mj: main jaws; pp: pseudo-phalangium; vjm: ventral jaw muscle; vpm: ventral pharyngeal muscles; z-b: Z-bands of the cross striated muscles of the ventral pharyngeal muscle.

Ventral of the trophi, lining the fibularium, several longitudinal fibres form a large ventral pharyngeal muscle plate (vpm, Figures 2A; 3A; 5C,D; 6A-C) and continues anteriorly as two small lateral muscle fibres. This ventral pharyngeal muscle plate is formed by 8-10 longitudinal cross striated muscle fibres (Figures 1B; 5C,D; 6A,C). The longest median muscle filament presents 8 z-bands (Figure 6A-C). However, even though the ventral pharyngeal muscle plate mostly underlies the fibularium, the ventral pharyngeal muscle is shifted more posteriorly compared to the fibularium. The plate is rounded at the lateral and posterior edges, hereby enveloping the trophi (including the fibularium) laterally and caudally (vpm: Figure 5C,D).

Dorsal to the fibularium, two pairs of muscles extend between the fibularium and the main jaws: one pair of lateral fibularium/main jaw muscles (lfm: Figures 5C; 6D-F), and one pair of median fibularium/main jaw muscles (mfm: Figures 5C; 6D-F). Both of them attach to the fibula caudalis of the fibularium. The lateral fibularium/main jaw muscle originates at the fibula caudalis (of the camera dorsalis 1), and supplies the anterior part of the main jaws. The median fibularium/main jaw muscle originates posterior of the fibula caudalis (of the camera dorsalis 1 and 2), and supplies a less anterior part of the main jaws than the lateral fibularium/main jaw muscle.

One pair of strong caudal muscles lines each cauda of the main jaws (cm: Figures 5D; 6D-F). They are thicker in their posterior parts where they follow the paired caudae of the main jaw. The contraction of this muscle moves the main jaws together.

Two short anterior fibularium/main jaw muscles (afm: Figure 6G-I) attach to the anterior part of the fibula lateralis at the camera lateralis, and link in this way the fibularium with the anterior parts of the main jaws.

Altogether, the anterior fibularium main jaw muscle, the lateral fibularium main jaw muscles, the median fibularium/main jaw muscles and the caudal muscle, are probably responsible for the opening of the main jaws and their previously described backward/forward movements (Kristensen and Funch [3]).

An unpaired very thin striated U-shaped dorsal jaw muscle (djm: Figure 6G-I) attaches at each extremity to the posterior ends of each dorsal jaw.

Lateral to the fibularium, one pair of strong cross striated ventral jaw muscles (vjm: Figures 5C,D; 6G-I) inserts at the posterior part of the accessory sclerite. They extend posterior of the trophi, attaching the sides of the fibularium and inserting posteriorly at the pharynx epithelium.

Anterior to the other parts of the trophi, two strong pharyngeal lamellae muscles (lm: Figures 5C,D; 6G-I) supply the accessory sclerites and the pharyngeal lamellae. The two pharyngeal lamellae muscles are very large and in the continuity of the paramedian ventral muscle and anterior lateral muscle. They enlarge dorso-ventrally at the terminal part. This observation confirms the supposed function of the pharyngeal lamellae (initially lamella oralis) as a supporting structure. This dorso-ventrally enlarged muscle could function in opening and closing the pharyngeal lamellae as a fan, affecting the volume of the pharynx. The ventral jaw muscle is probably functioning together with the pharyngeal lamellae muscle as an antagonist. Indeed, both muscles are connected to the accessory sclerite. When the pharyngeal lamellae muscles are contracted and the ventral jaw muscles relaxed, the pharyngeal lamellae will open and increase the volume of the pharynx cavity, also probably opening the mouth and allowing ventral jaws extrusion.

### Anti-Synapsin1 immunoreactivity

Anti-Synapsin 1 immunoreactivity (IR) was tested in ongoing studies of the nervous system (Bekkouche et al. unpublished) and surprisingly yielded a very distinct IR at the borders of the dorsal epidermis cells. This immunoreactivity, which is presented as spots along the borders, resembles the distribution pattern of the unique zip-junctions in *Limnognathia* (equivalents of adherens junctions) (Figure 2C). However this IR interpretation warrants further confirmation. Most importantly, the very distinct cell border signal has been proved useful in the present study for co-localizing the attachment sites of the dorso-ventral muscles. Thereafter it was possible, even in specimens not stained against Synapsin1, to retrieve the borders of the dorsal cells of the epidermis by increasing the brightness of the phalloidin stain (data not shown). The attachment of the last eight trunk dorso-ventral muscles to the dorsal epidermal cells could then be inferred in several specimens (Figure 4B). Furthermore, the synapsin 1 staining clearly shows that *Limnognathia maerski* has cell borders in the epidermis (as opposed to being syncytial) and therefore does not belong to Syndermata (Rotifera and Acanthocephala).

## Discussion

### Notes on the longitudinal musculature

In *L. maerski* most of the longitudinal musculature extends the entire body length, or at least the entire trunk, yet some muscles are restricted to certain areas, e.g., the adhesive ciliated pad (cpm: Figures 2A; 3A,C; 4A), the thorax (ldm: Figures 3A,C; 4B), the anterior part of the thorax (sav1,2: Figures 2A; 3A,C; 4A), etc. This repartition of the musculature supports functionally the separation of *L. maerski* into a head, a thorax and an abdomen. Similarly, in rotifers, many longitudinal muscles extend a subpart of the body, aiding the retraction of the foot or the corona [11,12]. Contrarily, most of the longitudinal muscles of Gnathostomulida extend the entire body length [9,10].

### Is the dorso-ventral musculature of *L. maerski* comparable to circular musculature?

The trunk dorso-ventral musculature of *L. maerski* (tdvm: Figures 2A,B; 3A,B,C; 4; 5A;B) superficially resembles the repeated incomplete circular muscles found in many rotifers. However, as described by Leasi and Ricci [12]: “the muscular system of rotifers generally consists of somatic and splanchnic (visceral) fibers. Somatic musculature is composed of two layers: an external layer made of separate circular rings and an internal layer of longitudinal muscles”. *Limnognathia maerski* lacks splanchnic fibers and the somatic musculature is only composed of longitudinal muscles. However, internal of these are found the dorso-ventral muscles. These are serially repeated along the lateral outline of the gut (tdvm: Figure 5A,B). The median position of the trunk dorso-ventral muscles, relative to the two pairs of lateral and paramedian ventral longitudinal muscles, does not conform to the somatic circular muscles found in rotifers, and homology of these muscles is unlikely. However, they can be functionally compared to those of rotifers: with lack of both outer and inner circular musculature, these dorso-ventral muscles may act both as a splanchnic musculature, aiding the movement of the food throughout the digestive system, as well as somatic dorso-ventral musculature, elongating the body during contraction. In rotifers, the incomplete circular muscles act as antagonists of the longitudinal musculature. When these somatic circular muscles contract, the pressure of the body fluids is redistributed and prompts the extension of the body [12]. The same function is assumed in *L. maerski* for the trunk dorso-ventral muscles. It is interesting to note the medio-ventral longitudinal muscles as they seem to extend at the same level as the ventralmost part of the trunk dorso-ventral muscles (tdvm and mvm: Figures 2A; 3A; 4; 5A,B). This suggests that the medio-ventral longitudinal muscles may specifically work as antagonists of the trunk dorso-ventral muscles in the same way as for rotifers.

Giribet et al. [5] propose, among other hypotheses, a relationship between Micrognathozoa and Cycliophora. In Cycliophora, inner dorso-ventral muscles are also present in the Pandora larva and the dwarf male life stages [33–35]. In the dwarf male, several sets of dorso-ventral muscles are present along the entire body length, while in the Pandora larva, only three pairs of dorso-ventral anterior muscles are present in addition to the incomplete circular muscles repeated through the entire body length. It is, though, difficult to establish any functional comparison with *L. maerski* since there is no gut present in these two cycliophoran stages.

Similar to *L. maerski*, dorso-ventral muscles are found internal of the longitudinal muscles in kinorhynchs [36]. Moreover, in the gastrotrich *Draculiciteria*, two sets of dorso-ventral muscles are found: one inside and one outside the longitudinal musculature, each supposed to be derived from splanchnic and somatic circular muscles, respectively [37]. The organization found in kinorhynchs can be compared to the attachment of the trunk dorso-ventral muscles to the epidermal cells containing the dorsal plates in *L. maerski*, even though the two conditions obviously are analogous. Additionally, in both kinorhynchs and *Draculiciteria*, as well as in rotifers, the contraction of the dorso-ventral musculature is supposed to be involved in the body extension [36,37].

This comparison between small sized pseudoceolomate or acoelomate animals, leads to the supposition that dorso-ventral muscles play a similar role as circular muscles, aiding the fluid circulation in the body and in *L. maerski*, possibly also changing the shape of the relatively large cells of the endodermis. Thus, the dorso-ventral muscle contractions possibly aid the movement of food particles in the gut, the vomit behavior, and the yet non-observed defecation. 

### Functional considerations of the pharynx musculature

#### Considerations on the jaw musculature of *L. maerski*

Six paired main elements are described in the jaws of *L. maerski*: i) The median basal plates ii) the large ventral fibularia, extending dorso-laterally, iii) the lateral-most ventral jaws, iv) the medio-dorsal main jaws, with posteriorly projecting caudae, v) the dorso-lateral dorsal jaws confined to the fibularium area, vi) and the antero-lateral pharyngeal lamellae [6]. For comparison we refer to the Table 1 that summarizes the various jaw homology hypotheses proposed in the literature between the Rotifera and *L. maerski*. A general consensus appears to exist for the homologies between the articularium and cauda of Gnathostomulida, the ramus and fulcrum of Rotifera and the main jaws and caudae of *L. maerski* [1,3,6,38].

**Table 1:** **Previously proposed homologies of** ***Limnognathia maerski*** **jaw parts and Rotifera jaw parts**

**Jaw elements in** ***Limnognathia maerski***	**Proposed homologies with rotifer trophi according to the authors**		
	**Kristensen and Funch** **[** 3 **]**	**De Smet [** 1 **]**	**Sørensen [** 6 **]**
Basal plates		Basal platelet (epipharynx)	Autapomorphy
Fibularium	Ramus	Manubrium + uncus	Autapomorphy
Ventral jaws	Uncus	Pseudomalleus (epipharynx)	Uncus
Accessory sclerites	Manubrium	Pseudomanubrium (epipharynx)	Manubrium
Main jaws dentarium	Ramus	Ramus	Ramus
Main jaws articularium	Fulcrum	Fulcrum	Fulcrum
Lamellae pharyngea	Epipharynx	Oral lamellae (epipharynx)	Epipharynx
Dorsal jaws	Autapomorphy	Pleural rod	Autapomorphy

No separate musculature associated to the basal plate in *L. maerski* has been found. Moreover, detailed examination of the ventral view of the SEM images of the jaws of *L. maerski* does not show any clear separation between the basal plates and the fibularium [1,6], suggesting that the basal plate could be an integrated part of the fibularium.

The dorsal jaw muscle apparently only connects the two dorsal jaws and is not attached to the pharyngeal wall. In Sørensen [6], the dorsal jaws are described as caudally attached to the internal side of the fibularia, possibly by a flexible ligament on each side, positioning the jaws in a 90° angle to the main jaws. A contraction of the dorsal jaw muscles would then pull apart the tips of the dorsal jaws, turning the jaws about 45° from their resting position.

The fibularium, as the most conspicuous jaw structure, is involved in the attachment of three out of eight jaw muscles systems, suggesting that the fibularium acts primarily as a supporting structure for the jaws and the pharynx, rather than an element directly implicated in the mastication. This assumption is consistent with the strong ventral pharyngeal muscle underlying the fibularium.

#### *Comparison of the pharyngeal musculature of* L. maerski *with those of other animals*

The ventral jaws and accessory sclerites of *L. maerski* make up as a functional unit that has been considered homologous with either the rotifer mallei [3,6] or the rotifer epipharynx [1] (see also Table 1). The ventral jaws can be moved independently and extruded through the mouth opening during foraging while the rest of the jaws are not. In rotifers, the different sclerites are more closely connected through ligaments, and the mallei cannot be fully protruded without also protruding parts of the incus as well (e.g., in *Bryceella stylata* [31] and *Dicranophorus forcipatus* [39]). In *L. maerski* no ligamentous connections exist between the ventral jaws and either the fibularium or main jaws, which allow the ventral jaws to move more independently from the other main elements of the jaw apparatus.

The ventral jaw muscle of *L. maerski* (vjm: Figure 5C,D; 6G-I) can be compared to the musculus circum-glandulis of Rotifera. This muscle connects the rami with other parts of the mallei [31,39,40]. Its ventral position, connection with the ramus and conspicuous shape, resembles the ventral pharyngeal muscle (conspicuous muscle made of several bundle) or the ventral jaw muscle (connection and position) in *L. maerski*. However, in rotifers this muscle is assumed to perform the spreading of the rami and eventually also the compression of the salivary glands [31], and such functions are not likely for the ventral jaw muscles in *L. maerski.* Hence, no equivalent of the ventral jaw muscle of *L. maerski* is found in Rotifera.

Underlying the fibularium, the conspicuous plate of the ventral pharyngeal muscle is present (vpm, Figures 2A; 3A; 5C,D; 6A-C). Composed of several longitudinal parallel muscles fibers, this structure is found neither in gnathostomulids nor rotifers. In Gnathostomulida though, a pharyngeal capsule is found, but it is formed by circular muscles enveloping the pharynx [27], which is structurally different from *L. maerski*. However, a strikingly similar ventral set of longitudinal muscles, encompassing two fanlike muscles forming a similar bowl, is found in the microscopic worm *Diurodrilus* (Spiralia *incertae sedis*) [30]. In *Diurodrilus*, this pharyngeal bowl also lines the pharynx ventrally, whereas its posterior part extends further dorsally compared to what is apparent in *L. maerski*. In L. maerski, the configuration of the muscle plate indicates that it is implicated in the extrusion and sinking movements of the fibularium and possibly causes changes in the volume of the pharyngeal cavity.

Functionally, this muscle could also be similar to the mastax receptor retractor found in the rotifer *Pleurotrocha petromyzon* as well as other rotifers with virgate mastax [40], aiding the total movement of the mastax by changing the shape of the pharynx cavity. However, the rotifer mastax receptor retractors are located dorsal to the jaw, which makes an actual homology with the micrognathozoan ventral pharyngeal muscle unlikely. We assume a similar function of the ventral pharyngeal muscle in *L. maerski*, which when contracting seems to move the entire jaws system, during the so-called vomit behavior. Morphologically, the similarity of the plate-bowl-shaped ventral pharyngeal muscle of *L. maerski* and *Diurodrilus* is striking [30] and not found in Rotifera and Gnathostomulida.

The main jaws represent the central element of the micrognathozoan jaw apparatus, and there is a consensus about homologizing the main jaws with the rotifer incus [1,3,6] (see also Table 1). Two different sets of main jaw muscles connect the main jaws with other sclerites or with the pharyngeal wall. The first set, related to the fibularium, is a “lateral connection” created by the anterior fibularium main jaw muscle, the lateral fibularium main jaw muscle and the median fibularium main jaw muscle. The second one, independent of the fibularium, is a “posterior connection” created by the caudal muscle. In *L. maerski*, the “lateral connection” is the most prominent in the main jaws and it is operated by 3 sets of muscles (anterior fibularium main jaw muscle, lateral fibularium main jaw muscle, median fibularium main jaw muscle, respectively afm, lfm, mfm: Figure 6D-I). In Gnathostomulida, the lateral connection is also dominant, realized by the diductor muscles [9,26] which do not connect to a lateral sclerite but to the dorsal wall of the pharynx. In *L. maerski*, the fibularium has the function of attaching the muscles involved in the lateral connection. Among rotifers, sparse examples of lateral connections can be found. The only muscle having this arrangement is the musculus ramo-manubricus found in *Filinia longiseta* [41] and *Trichocerca rattus* [33], both having very peculiar trophi (respectively malleoramate and asymmetrical virgate). In Rotifera, though, the posterior connection is well documented in the abundant work of the series of confocal and TEM studies by the Ahlrichs Group [31,32,39–41], who refers to this muscle as the musculus fulcro ramicus. Furthermore, Riemann and Ahlrichs, emphasize the wide repartition of this muscle within Rotifera, suggesting the homology of this muscle across the taxon [39]. Then, the cauda muscle of *L. maerski* (cm: Figure 6D-F) could also be homologous to the musculus fulcro ramicus of Rotifera. A difference between those two muscles is that the cauda muscle seems to embed, or at least extend closely the cauda, while the musculus fulcro ramicus is more diagonal in its orientation. Additionally, the cauda muscle goes more posterior and seems to insert in the pharyngeal wall, while the musculus fulcro-ramicus is posteriorly restricted to the fulcrum.

Only muscles functionally implicated in the opening of the main jaws (not in the closing) have been found in *L. maerski*. As proposed for Rotifera and Gnathostomulida, we assume that the kinetic energy release of the cuticular parts provokes a passive closing of the pincer like sclerites in *L. maerski* [26,27,39].

## Conclusions

Due to its simplicity, the longitudinal musculature of *L. maerski* is only roughly comparable to the musculature of other groups. However, the dorso-ventral musculature shows a functional similarity to the semi-circular muscles of the closely related Rotifera and other meiofaunal animals.

With regards to the pharyngeal musculature, only one specific homology between the cauda muscle of *L. maerski* and the musculus fulcro ramicus of rotifers can be hypothesized. However, the functional and morphological comparisons of the jaw musculature among gnathiferans aid the understanding of how such small complex systems can be moved. Two different “strategies” can be observed in the jaw apparatus of Rotifera versus Gnathostomulida: in rotifers, sclerites are moved by muscles connected to other jaw parts whereas in gnathostomulids the less complex jaws are moved by muscles connected directly to the pharyngeal wall. It is not surprising considering the complexity of the jaws of *L. maerski* that the jaw musculature and function are more comparable to that of Rotifera. However, the independence of the ventral jaw of *L. maerski* relative to the rest of the trophi is an interesting difference between *L. maerski* and Rotifera. Additionally, the striking similarity between the ventral pharyngeal muscle of Micrognathozoa and the pharyngeal bowl-shaped muscle of *Diurodrilus* is interesting in relation to the debated close relationship between the jaw-less *Diurodrilus* and Micrognathozoa [3,30].

Several functional analogies and common patterns could be shown between *L. maerski* and other Gnathifera or small sized animals, but the systematic value of the musculature of *L. maerski* still appears quite limited. However, further studies are needed in Gnathifera. De Smet [1] emphasizes the poor knowledge of the epipharynx of Rotifera. For example, Riemann and Ahlrichs [39], in their study on *Dicranophorus forcipatus* cannot assign any clear function to the hypopharyngeal elements. Furthermore, no complete detailed studies of the musculature and function of trophi of the Seisonidae, Bdelloidea (both Rotifera) and Filospermoidea (Gnathostomulida) have been done so far. Nevertheless, a systematic comparison will still be challenging since the trophi of Bdelloidea and Seisonidea are very modified, and the jaws of Filospermoidea have a relatively simple pincer-like structure, such as in *Haplognathia*.

## Material and methods

### Collection of specimens

Specimens used for TEM were part of the original material that were collected at the type locality in the Isunngua Spring on Disko Island, West Greenland, 69°43'N 51° 56'W, and used for the description of Micrognathozoa [3]. Specimens for CLSM were collected in July-August 2010 and 2013 at the same locality.

### Transmission electron microscopy

Specimens were fixed in trialdehyde 8% (after Kalt and Tandler [42] and Lake, [43], without acrolein) and postfixed in 1% osmium-tetroxide with 0.1M sodium cacodylate buffer for 1 hour (h) at 20°C. Specimens were then dehydrated through an ethanol series, transferred to propylene oxide, and embedded in epoxy resin type TAAB 812®. Ultrathin serial sections were stained with uranyl acetate and lead citrate [44]. TEM examinations were performed with a JEOL JEM 100SX transmission electron microscope.

### Cytochemistry and CLSM

Specimens of *L. maerski* were fixed for 2 h at room temperature (or overnight at 4°C) in 2% paraformaldehyde in 0.15M phosphate buffered saline (PBS), pH 7.4, rinsed and stored in PBS plus 0.05% NaN_3_. Entire specimens were preincubated two hours in PTA (PBS with 0.5% Triton-X, 0.05% NaN_3_, 0.25% bovine serum albumin (BSA) and 5% sucrose) and afterwards incubated for 2h at room temperature in 0.34 μM Alexa fluor 488 phalloidin (Invitrogen, A12379) in PTA and finally mounted in Vectashield® (Vector Laboratories, Burlingame, CA) containing DAPI. For immunostaining against synapsin1, specimens were preincubated two hours in PTA and incubated for 12h at room temperature with antibodies anti synapsin1 raised in Rabbit (ENZO life Sciences, ADI-VAS-SV061-E). Then the specimens were rinsed in PBS, pre-incubated 2h in PTA and incubated 12h at room temperature with the secondary antibody anti-rabbit, conjugated with the fluorophore FITC (SIGMA, prod. num. f0382). Finally the specimens were rinsed in PBS and mounted in Vectashield®. Preparations were analyzed with an Olympus Fluoview FV1000 CLSM or a Leica TCS SP5 CLSM. The specificity of the antibodies was tested by examining specimens where each of the primary and secondary antibodies were omitted.

### Image treatment

Z-stacks or parts of them of CLSM files were projected into 2D-images (MIP images = maximum intensity pixel images) and 3D iso-surface reconstructed in Imaris v7 (Bitplane AG, Zürich, Switzerland). Depth coded Z-stack images of F-actin staining are also presented (Leica imaging software), were the depth-gradient follows the area of the spectral light with the uppermost structures appearing red, and the more distant one blue. Free hand drawings and plate setups were done with Adobe Illustrator CS6 and Image modification done with Adobe Photoshop CS6.
